# Quantitative analysis of crystallinity in an argyrodite sulfide-based solid electrolyte synthesized *via* solution processing†

**DOI:** 10.1039/c9ra00949c

**Published:** 2019-05-08

**Authors:** So Yubuchi, Hirofumi Tsukasaki, Atsushi Sakuda, Shigeo Mori, Akitoshi Hayashi, Masahiro Tatsumisago

**Affiliations:** Department of Applied Chemistry, Graduate School of Engineering, Osaka Prefecture University 1-1, Gakuen-cho, Naka-ku Sakai Osaka 599-8531 Japan hayashi@chem.osakafu-u.ac.jp tatsu@chem.osakafu-u.ac.jp; Department of Material Science, Graduate School of Engineering, Osaka Prefecture University 1-1, Gakuen-cho, Naka-ku Sakai Osaka 599-8531 Japan

## Abstract

Liquid-phase synthesis is a useful technique for preparing argyrodite sulfide-based solid electrolytes, and the synthesis conditions such as heat treatment strongly affect the conductivity. Because the understanding of structural changes reveals crucial information about their properties, it is necessary to evaluate this change during heat treatment to determine the factors that affect the conductivity. In this study, X-ray diffraction measurements and transmission electron microscope observations reveal the effects of heat treatment on the crystallinities and ionic conductivities in the synthesis process of argyrodite electrolytes with tetrahydrofuran and ethanol. The amorphous material is in the main phase when heated at low temperatures below 200 °C and exhibits relatively low conductivities of *ca.* 2 × 10^−4^ S cm^−1^ despite precipitation of the argyrodite crystals. As the heat treatment temperature increases, the ratio of argyrodite crystals increases, involving nucleation and grain growth, leading to high conductivities of over 10^−3^ S cm^−1^. It is critical to control the ratio of the amorphous and crystal phases to achieve high conductivities in the synthesis of argyrodite electrolytes *via* liquid-phase processing.

## Abstract 2

Liquid-phase synthesis is a useful technique for preparing argyrodite sulfide-based solid electrolytes, and the synthesis conditions such as heat treatment strongly affect the conductivity.

## Graphical abstract

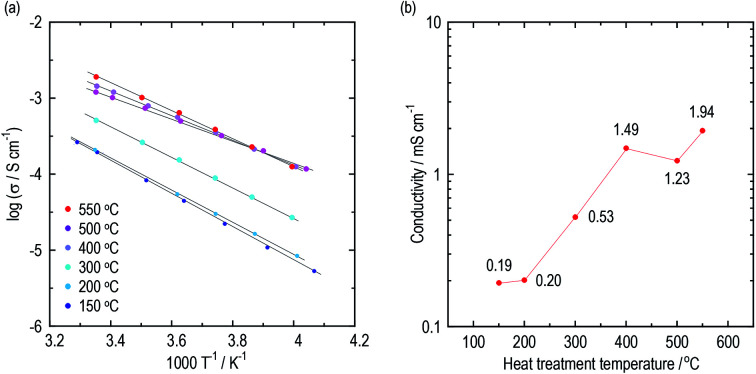

## 1. Introduction

Solid electrolytes are indispensable for achieving high energy densities, high power densities, and long lifetimes in all-solid-state batteries. Recently, sulfide-based solid electrolytes with high ionic conductivities and favorable mechanical properties have been focused on as a practicable material. Up until now, Li_10_GeP_2_S_12_,^1,2^ Li_7_P_3_S_11_,^3,4^ argyrodite,^5–8^ and Na_3_PS_4_ (ref. 9 and 10) sulfide-based solid electrolytes with conductivities of 10^−3^ to 10^−2^ S cm^−1^ have been developed, realizing near-future applications of all-solid-state batteries.

A liquid-phase process for preparing the sulfide-based solid electrolytes is a necessary technique to replace conventional solid-phase and vapor-phase methods. Based on the initial report on β-Li_3_PS_4_ synthesized using tetrahydrofuran (THF),^11^ there have been several investigations of the liquid-phase syntheses of various solid electrolytes such as β-Li_3_PS_4_,^12–14^ Li_7_P_3_S_11_,^15–18^ Li_7_P_2_S_8_I,^19,20^ Li_6_PS_5_X (X = Cl, Br, I, BH_4_),^21–26^ and Na_3_MS_4_ (M = P, Sb).^27–33^ In particular, we have focused on the liquid-phase synthesis of argyrodite electrolytes *via* solutions because of their high applicability. By using bi-solvents of THF and ethanol (EtOH), argyrodite electrolytes were synthesized through a simple solution process. The electrolyte showed a high lithium-ion conductivity of 3.1 mS cm^−1^ at 25 °C.^23^ In addition, coating the electrode active material particles with electrolytes and infiltrating the electrolytes into the porous electrode sheets using the precursor solutions were useful for obtaining dense composite electrodes with wide electrode–electrolyte contact areas, achieving high-performing all-solid-state cells.^21–24,34,35^ These results are sufficient for demonstrating the advantages of the liquid-phase techniques for all-solid-state cells. To obtain a deeper understanding of liquid-phase synthesis, the reaction mechanisms in the solution and the structural changes after removing the solvents must be clarified. Although we have revealed that it is important for the precursor solutions to be basic and not contain P–S–P bonds in the synthesis process of argyrodite electrolytes,^23^ it is also necessary to clarify the effect of heat treatment on their ionic conductivities.

Herein, we investigated the relationship among the heat treatment temperatures, crystallinities, and ionic conductivities of Li_6_PS_5_Br argyrodite electrolytes synthesized *via* solutions using X-ray diffraction (XRD) measurement and transmission electron microscope (TEM) observation. Because lower heat treatment temperatures caused an amorphous component to have higher volume ratios than the argyrodite crystal, the electrolytes exhibited relatively low conductivities. The volume ratios of argyrodite crystals increased with nucleation and grain growth, accompanied by solid-state reactions, as the heat treatment temperatures increased, resulting in high conductivities of 10^−3^ S cm^−1^ or more. The ratio of the amorphous and crystal components was found to vary significantly in the liquid-phase-synthesized electrolyte with the variation the heat treatment temperature, which greatly affected the conductivity.

## 2. Results and discussion

### 2.1 Ionic conductivity measurement

**Fig. 1:**
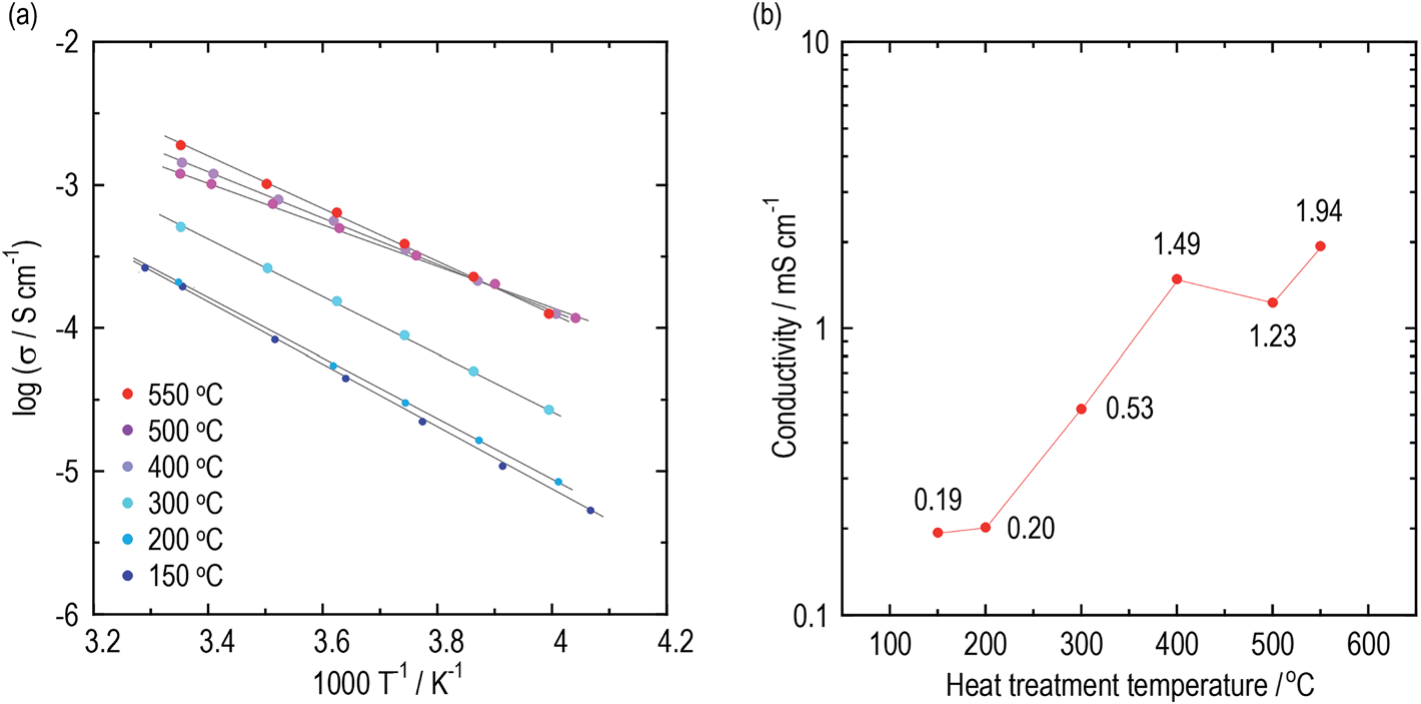
Conductivities of the argyrodite electrolytes prepared by the liquid-phase technique. (a) Arrhenius plots and (b) the conductivities at 25 °C of the argyrodite electrolytes. Green compacts pressed at 720 MPa were used.

Conductivities of the argyrodite electrolytes were evaluated by electrochemical impedance measurements. Argyrodite electrolytes were synthesized in the same manner as reported previously.^23^Fig. 1(a) and (b) show Arrhenius plots and the conductivities at 25 °C of the electrolytes heated at various temperatures, respectively. Green compacts pressed at 720 MPa were used for the measurements. Fig. S1† shows the Nyquist plots of the electrolytes prepared by heating at 150 °C and 400 °C. One semicircle was observed for each electrolyte even at the low temperature region, and thus the contribution of the grain and grain boundary is not distinguished. The electrolytes heated at 150 °C and 200 °C exhibit almost the same conductivities of *ca.* 2 × 10^−4^ S cm^−1^, which is close to those of Li_2_S–P_2_S_5_ glasses.^36,37^ As the heat treatment temperatures increase, the conductivities dramatically increase. The electrolytes exhibit conductivities of 1 mS cm^−1^ or more when heated at 400 °C and higher. The electrolyte heat-treated at 550 °C exhibits a maximum conductivity of 1.9 mS cm^−1^ at 25 °C, comparable to that of Li_6_PS_5_Br prepared by the solid-phase method. The activation energies gradually decrease with the increase in heat treatment temperature up to 500 °C (Table 1). To obtain a deeper understanding of the changes in conductivities and activation energies with heating temperature, the argyrodite electrolytes were evaluated using XRD measurements and TEM observations.

**Table 1:** Conductivities and activation energies of the argyrodite electrolytes. Green compacts pressed at 720 MPa were used

HT temperature/°C	150	200	300	400	500	550
Conductivity at 25 °C/mS cm^−1^	0.19	0.20	0.53	1.49	1.23	1.94
Activation energy/kJ mol^−1^	42.3	40.3	38.3	31.2	27.7	34.9

### 2.2 X-ray diffraction measurement

**Fig. 2:**
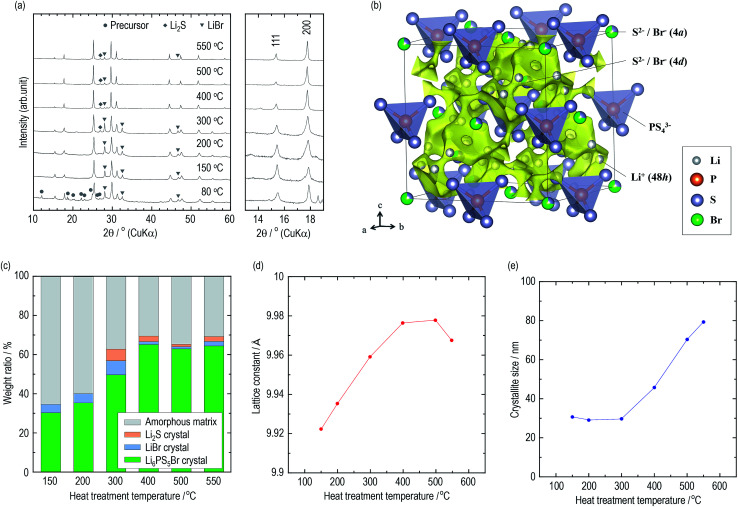
Structural analysis of the argyrodite electrolytes using XRD measurement. (a) XRD patterns of the argyrodite electrolytes. (b) Crystal structure and three-dimensional bond-valence-sum map isosurfaces of Li_6_PS_5_Br heated at 400 °C with the outlined unit cell. The Li, P, S, and Br sites are represented by gray, orange, purple, and green balls, respectively. Yellow isosurfaces have been obtained from the bond valence technique (v.u. ±0.25). (c) Weight ratios of the constituents in the argyrodite electrolytes. (d) Lattice constants and (e) the crystallite sizes of Li_6_PS_5_Br crystals.

The constituents of the electrolytes were elucidated by XRD measurements. Fig. 2(a) presents XRD patterns of the argyrodite electrolytes prepared by drying and heating at various temperatures. In the electrolyte dried at 80 °C, unknown diffraction peaks are observed, as well as diffraction peaks attributed to the argyrodite and LiBr crystals. Similar peaks are also detected when the argyrodite electrolytes are dissolved into and precipitated from an EtOH solvent (Fig. S2†), suggesting that the product is a precursor consisting of argyrodite crystals and EtOH molecules. The electrolytes heated at 150 °C and 200 °C mainly consist of argyrodite crystals with a small amount of LiBr crystals, indicating that the precursor decomposed and completely removed the solvent. When heating the electrolytes over 300 °C, the Li_2_S crystal precipitates in addition to the argyrodite crystal and LiBr crystal. Heat treatment at 400 °C leads to a lower intensity of diffraction peaks of the LiBr crystal and Li_2_S crystal. The XRD measurements provided the following four qualitative results. The increase in heat treatment temperatures resulted in (1) the decrease in intensities of the halo patterns originating from the amorphous material, (2) sharper diffraction peaks of the argyrodite crystal, (3) the change in the peak intensities corresponding to 111 and 200 reflection indices, and (4) fewer impurities such as Li_2_S crystals and LiBr crystals. To obtain quantitative results, Rietveld analysis was performed.

Crystallographic phase identification was performed using powder XRD measurement and Rietveld analysis.^38,39^ It can be confirmed that all the electrolytes heated at temperatures ranging from 150 °C to 550 °C possess argyrodite structures (cubic, *F*4̄3*m*, (216)) from the powder XRD patterns and Rietveld refinement analysis (Fig. S3†). The structural parameters are listed in Tables S2–S7.†Fig. 2(b) presents the crystal structure and bond-valence-sum (BVS) map isosurfaces (v.u. ±0.25) of the Li_6_PS_5_Br crystal heated at 400 °C, which had a high conductivity of 1 mS cm^−1^ or more. The S^2−^ and Br^−^ anions form a face-centered cubic sub-lattice (Wyckoff positions 4a and 4d) and PS_4_^3−^ tetrahedra (P on Wyckoff position 4b). The S^2−^ and Br^−^ anions randomly occupy the 4a and 4d sites because of their similar ionic radii. The Li^+^ ion site technique indicates that a cage-like conduction pathway of lithium ions formed in three dimensions, leading to high conductivities of the Li_6_PS_5_Br crystals.

Based on the structural parameters obtained from the Rietveld analysis, the constituents in the electrolytes were evaluated using a whole powder pattern fitting (WPPF) technique with the Al_2_O_3_ crystal, which is a typical reference material. Mixtures of the electrolyte and Al_2_O_3_ crystal in a weight ratio of 50 : 50 were prepared by calculating the amount ratios of the amorphous phase in the electrolytes. XRD patterns and Rietveld refinement analysis results of the mixture are also presented in Fig. S4 and S5,† respectively. Fig. 2(c) and Table 2 present the weight ratios of the argyrodite crystal, LiBr crystal, Li_2_S crystal, and amorphous material in the electrolytes heated at various temperatures. Interestingly, the argyrodite crystal and LiBr crystal only account for 30.3 and 4.3 wt% in the electrolyte heated at 150 °C, respectively, indicating that the electrolyte primarily contained Li–P–S–Br amorphous components. It is known that gas generation, which is derived from the attached solvents, forms the sulfide-based materials in the amorphous state during the thermal decomposition of the precursor in the synthesis using organic solvents.^15,40^ Similar phenomena are considered to have occurred by decomposing the argyrodite precursor solvated by THF and/or EtOH. The heat treatments over 300 °C precipitated the LiBr crystal and Li_2_S crystal as impurities. However, the heat treatments over 400 °C decrease the number of impurities and increase the weight ratio of the argyrodite crystal to *ca.* 65 wt%. In addition, the lattice constants of the argyrodite crystals gradually increase as the heat treatment temperatures are raised (Fig. 2(d)). Considering that the radius of Br^−^ ions (1.96 Å) is larger than that of S^2−^ ions (1.84 Å), these results suggest that the LiBr crystal was incorporated into the argyrodite crystal by the solid-state reaction. Interestingly, the amorphous phase remain at proportions of more than 30 wt% in every electrolyte heated at every temperature.

**Table 2:** Weight ratios of the constituents in the argyrodite electrolytes calculated from a WPPF technique

HT temperature/°C	150	200	300	400	500	550
Li_6_PS_5_Br crystal	30.3	35.4	49.7	65.2	63.0	64.5
LiBr crystal	4.3	4.6	7.2	1.5	1.1	2.3
Li_2_S crystal	0	0	6.0	2.8	1.3	2.5
Amorphous matrix	65.4	43.1	37.1	30.5	34.6	20.7

Fig. 2(e) presents the average crystallite sizes calculated from the diffraction peaks of the 220, 311, and 222 reflection indices of the argyrodite crystals. The heat treatments below 300 °C promote nucleation of the argyrodite crystals. As the heat treatments increase above 400 °C, the crystallite sizes increase with the grain growth.

### 2.3 Transmission electron microscope observation

**Fig. 3:**
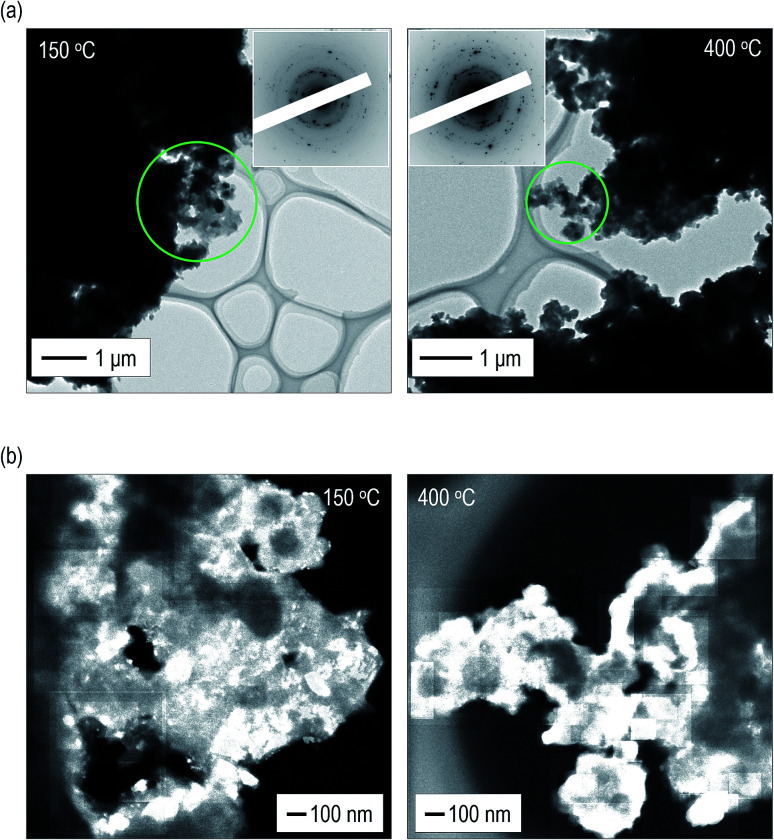
Microstructure observation of the argyrodite electrolytes using TEM observation. (a) BF images with ED patterns and (b) superposed DF images of the argyrodite electrolytes heated at 150 °C and 400 °C.

To obtain a deeper understanding and mutual validation using XRD measurements, the volume ratio of the crystals was directly evaluated using *ex situ* TEM observations. The electrolytes heated at 150 °C and 400 °C were used. Fig. 3(a) shows bright field (BF) images with electron diffraction (ED) patterns obtained from the observation areas marked by green circles. Strong diffraction spots are observed in the ED patterns of the electrolytes. By constructing the intensity profiles from the ED patterns,^41–45^ the main crystalline phase was identified as the argyrodite crystal, which is consistent with the results of the XRD measurements (Fig. S6†). Fig. S7† shows dark field (DF) images of the electrolytes heated at 150 °C and 400 °C. Note that the bright–contrast regions are different in each DF image. Fig. 3(b) presents the images taken by superposing all the DF images.^46,47^ The bright–contrast regions corresponding to the diffraction spots are visualized, enabling the spatial distribution of the crystallite to be clearly observed. The crystallite size precipitated in the electrolyte heated at 400 °C is larger than that heated at 150 °C. Moreover, it can be identified that large crystallites are connected to each other and the number of grain boundaries decreases in the electrolyte heated at 400 °C. From the superimposed DF images, the volume ratio of the crystallized region was calculated. The ESI† shows the detailed calculation procedure. The volume ratios of the crystals are *ca.* 32 and 62 vol% in the electrolytes heated at 150 °C and 400 °C, respectively. These values are in good agreement with those obtained from the XRD measurement.

### 2.4 Effect of heat treatment

**Fig. 4:**
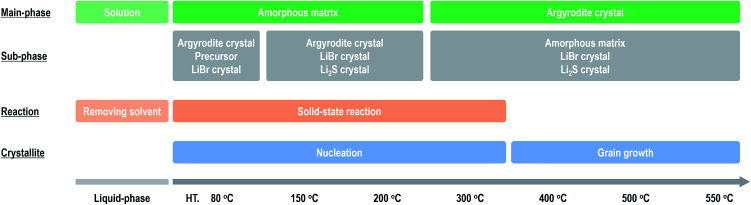
Effect of heat treatment on the formation mechanism and ionic conductivities of the argyrodite electrolytes prepared *via* liquid-phase processing.

Fig. 4 summarizes the effect of heat treatment at various temperatures on the argyrodite electrolytes synthesized by the liquid-phase technique. When drying the solution at room temperature, a precursor consisting of argyrodite and EtOH is formed. Subsequent heat treatments below 200 °C lead to the formation of amorphous components in the electrolytes. Therefore, the electrolytes exhibit relatively low conductivities of *ca.* 2 × 10^−4^ S cm^−1^ at 25 °C despite the precipitation of the argyrodite crystals. As the heat treatment temperatures increase from 200 °C to 400 °C, the ratios of argyrodite crystals increase to *ca.* 65 wt% because of the crystallization involving the solid-state reactions. The electrolytes heated at over 400 °C exhibit conductivities of over 10^−3^ S cm^−1^. In other words, the argyrodite electrolytes with high conductivities were prepared by the two-step process of the liquid-state reaction, forming the precursor that mainly includes the amorphous matrix and the subsequent solid-state reaction between the precursor and residual components. Moreover, the conductivities gradually improve by heat treatments at higher temperatures because of the growing crystallite sizes. Interestingly, the argyrodite electrodes exhibit high conductivities of 10^−3^ S cm^−1^ although the amorphous components remain at ratios of more than 30 wt% in all the electrolytes heated at any temperature. These results indicate that the increase in the proportions of argyrodite crystals in the electrolytes by controlling the nucleation and grain growth processes is important for increasing the ionic conductivities of the electrolytes synthesized *via* liquid-phase processing. The compositional analyses will be done for Rietveld refinement to determine more accurate structure.

## 3. Conclusions

In conclusion, we revealed the effects of heat treatment on the ionic conductivities of argyrodite sulfide-based solid electrolytes synthesized *via* liquid-phase processing. With simple XRD measurements and TEM observations, it was possible to quantify the fractions of not only the argyrodite crystal but also the amorphous matrix in the electrolytes. The amorphous matrix strongly affected the ionic conductivity. In particular, when heated at low temperatures below 200 °C, where various techniques such as coating and infiltrating can be applied to all-solid-state batteries, the volume ratio of the amorphous phase was large, suppressing the ionic conduction of the argyrodite electrolyte. Enhancing the crystallinity by optimizing the synthetic conditions such as selecting solvents with less steric hindrances and controlling the heat treatment process is very critical for achieving higher conductivities of the argyrodite electrolytes synthesized by the liquid-phase technique.

## 4. Experimental section

### 4.1 Material synthesis

Argyrodite Li_6_PS_5_Br solid electrolytes were synthesized by a multi-step liquid-phase technique based on our previous report.^23^ Li_2_S (Mitsuwa Chemical Co. Ltd., 99.9%), P_2_S_5_ (Aldrich, 99%), and LiBr (Aldrich, 99.9%) were used as the starting materials. The synthesis was conducted *via* the following four steps: (1) Li_2_S and P_2_S_5_ with a stoichiometry of 3 to 1 were mixed in super-dehydrated tetrahydrofuran without a stabilizer (THF, Wako). The mixture was stirred overnight. A THF suspension containing the Li_3_PS_4_·3THF precursor was thus obtained. (2) Li_2_S and LiBr (1 : 1 molar ratio) were dissolved in super-dehydrated ethanol (EtOH, Wako, 99.5%). (3) The THF suspension and EtOH solution were mixed to obtain a pale green THF–EtOH precursor solution of Li_6_PS_5_Br. The final molar ratio of Li_2_S, P_2_S_5_, and LiBr was 5 : 1 : 2 in the stoichiometric proportion of Li_6_PS_5_Br. The concentration of Li_6_PS_5_Br in the THF–EtOH precursor solution was 4.5 wt%. (4) The precursor solution was dried at 80 °C and 150 °C under vacuum for 3 h to generate a solid powder. Further heat treatments at 200 °C, 300 °C, 400 °C, 500 °C, and 550 °C under Ar gas flow were performed.

To identify the precursor of Li_6_PS_5_Br, Li_6_PS_5_Br prepared by a mechanochemical (MC) technique^6^ was dissolved into and precipitated from the EtOH solvent. The MC treatment was carried out using a planetary ball mill (Pulverisette 7, Fritsch) with a zirconia pot (45 mL in volume) and 15 zirconia balls (10 mm in diameter) at ambient temperature. The rotational speed was set at 600 rpm and the milling time was 45 h. The obtained Li_6_PS_5_Br was dissolved into EtOH. After that, solid powder was obtained by drying at room temperature under a dry Ar atmosphere.

### 4.2 Ionic conductivity measurement

Ionic conductivities of the electrolytes were measured by electrochemical impedance spectroscopy. The data were collected in the range from 10 Hz to 1 MHz using an impedance analyzer (1260, Solartron Analytical), with an applied AC voltage of 10–25 mV. To determine the ionic conductivities, green compacts were prepared by pressing at 720 MPa at room temperature. Au current collectors were used. The pellet was sealed in a laminate-type pouch cell to prevent exposure of the electrolytes to air. The temperature dependences of the conductivities were measured from the low-temperature region of about −30 °C. The temperatures were controlled with an EtOH-cooled bath and constant-temperature bath. The cells were held at the specified temperature for 2 h to allow the system to reach thermal equilibrium. Lithium-ion migration activation energies were calculated from the slopes of the Arrhenius plots:*σ* = *σ*_0_ exp(−*E*_a_/*RT*)where *σ* is the ionic conductivity, *T* is the absolute temperature, *σ*_0_ is the pre-exponential factor for ionic conduction, *E*_a_ is the activation energy for ionic conduction, and *R* is the gas constant.

### 4.3 X-ray diffraction measurement and Rietveld analysis

Crystallographic phase identification was performed using an X-ray diffractometer (SmartLab, Rigaku) with Cu-Kα radiation. Diffraction data were collected in steps of 0.02° over a 2*θ* range of 5–80° at a scan rate of 4° min^−1^. XRD measurements were conducted using an airtight container with a beryllium window to prevent exposure of the electrolytes to air. The computer program RIETAN-FP^38^ was used for the Rietveld analysis. Visualization of the crystal model was carried out using the VESTA software.^48^ The lithium-ion migration pathway in the crystal structure was analyzed by bond-valance-sum calculation using the computer program PyAbstantia and RIETAN-FP.^38^

For evaluating the constituents in the electrolytes, whole powder pattern fitting (WPPF) analysis was conducted using the computer program PDXL2 (Rigaku Co.).^39^ An Al_2_O_3_ crystal was used as a reference material. The analysis was conducted *via* the following four steps: (1) structural parameters of the electrolytes and Al_2_O_3_ crystal were obtained from Rietveld analysis. (2) Mixtures of the electrolyte and Al_2_O_3_ crystal at a weight ratio of 50 : 50 were prepared and XRD patterns were then obtained. (3) Weight ratios of the Al_2_O_3_ crystal and each crystal were calculated by the WPPF technique. Note that the structural parameters of the electrolytes and Al_2_O_3_ crystal were fixed. (4) Weight ratios of the amorphous crystal were calculated according to the following formula.*W*_amorphous_ = *W*_alumina_ − (*W*_argyrodite_ + *W*_LiBr_ + *W*_Li_2_S_)

### 4.4 Transmission electron microscope observation

The microstructure observation and volume ratio calculation of the crystals were performed using a TEM system (JEM-2100, JEOL) with an accelerating voltage of 200 kV. The sample was mounted on an amorphous carbon film supported by a Cu grid, which was attached to a double-tilt vacuum TEM holder (model 648, Gatan) in a dry Ar glove box. The pressure in the TEM equipment was *ca.* 1.0 × 10^−5^ Pa. Bright field (BF) images, dark field (DF) images, and electron diffraction (ED) patterns were obtained. The ED patterns were transformed into a one-dimensional intensity profile by the computer program “ProcessDiffraction.”^42–45^ The amount ratios of the crystals were calculated *via* the following four steps: (1) ED patterns from the argyrodite crystal regions were obtained, which showed Debye rings. (2) DF images were taken of all the diffraction spots forming the Debye rings. (3) The regions corresponding to the diffraction spots were visualized as bright–contrast regions by superposing all the DF images. (4) The areas with a luminance of 240 or more were regarded as crystallized regions, and the pixel number was then integrated using the image processing software “Image J” to calculate the volume ratio of the crystals. Note that calculations were performed excluding the vacuum region and thick electrolyte region (Fig. S8†).

## Conflicts of interest

There are no conflicts to declare.

## Supplementary Material

RA-009-C9RA00949C-s001

## References

[cit1] Kamaya N., Homma K., Yamakawa Y., Hirayama M., Kanno R., Yonemura M., Kamiyama T., Kato Y., Hama S., Kawamoto K., Mitsui A. (2011). Nat. Mater..

[cit2] Kato K., Hori S., Saito T., Suzuki K., Hirayama M., Mitsui A., Yonemura M., Iba H., Kanno R. (2016). Nat. Energy.

[cit3] Seino Y., Ota T., Takada K., Hayashi A., Tatsumisago M. (2014). Energy Environ. Sci..

[cit4] Ujiie S., Hayashi A., Tatsumisago M. (2014). Materials for Renewable and Sustainable Energy.

[cit5] Deiseroth H.-J., Kong S.-T., Eckert H., Vannahme J., Reiner C., Zaiβ T., Schlosser M. (2008). Angew. Chem., Int. Ed..

[cit6] Boulineau S., Courty M., Tarascon J.-M., Viallet V. (2012). Solid State Ionics.

[cit7] Deiseroth H.-J., Maier J., Weichert K., Nickel V., Kong S.-T., Reiner C. (2011). Z. Anorg. Allg. Chem..

[cit8] Kraft M. A., Culver S. P., Calderon M., Böcher F., Krauskopf T., Senyshyn A., Dietrich C., Zevalkink A., Janek J., Zeier W. G. (2017). J. Am. Chem. Soc..

[cit9] Hayashi A., Noi K., Sakuda A., Tatsumisago M. (2012). Nat. Commun..

[cit10] Hayashi A., Noi K., Tanibata N., Nagao M., Tatsumisago M. (2014). J. Power Sources.

[cit11] Liu Z., Fu W., Payzant E. A., Yu X., Wu Z., Dudney N. J., Kiggans J., Hong K., Rondinone A. J., Liang C. (2013). J. Am. Chem. Soc..

[cit12] Wang H., Hood Z. D., Xia Y., Liang C. (2016). J. Mater. Chem. A.

[cit13] Phuc N. H. H., Totani M., Morikawa K., Muto H., Matsuda A. (2016). Solid State Ionics.

[cit14] Phuc N. H. H., Morikawa K., Totani M., Muto H., Matsuda A. (2017). Ionics.

[cit15] Ito S., Nakakita M., Aihara Y., Uehara T., Machida N. (2014). J. Power Sources.

[cit16] Calpa M., Rosero-Navarro N. C., Miuram A., Tadanaga K. (2017). RSC Adv..

[cit17] Xu R. C., Wang X. L., Zhang S. Z., Xia Y., Xia X. H., Wu J. B., Tu J. P. (2018). J. Power Sources.

[cit18] Wang Y., Lu D., Bowden M., Khoury P. Z. E., Han K. S., Deng Z. D., Xiao J., Zhang J.-G., Liu J. (2018). Chem. Mater..

[cit19] Rangasamy E., Liu Z., Gobet M., Pilar K., Sahu G., Zhou W., Wu H., Greenbaum S., Liang C. (2015). J. Am. Chem. Soc..

[cit20] Phuc N. H. H., Hirahara E., Morikawa K., Muto H., Matsuda A. (2017). J. Power Sources.

[cit21] Yubuchi S., Teragawa S., Aso K., Tadanaga K., Hayashi A., Tatsumisago M. (2015). J. Power Sources.

[cit22] Yubuchi S., Uematsu M., Deguchi M., Hayashi A., Tatsumisago M. (2018). ACS Appl. Energy Mater..

[cit23] Yubuchi S., Uematsu M., Hotehama C., Sakuda A., Hayashi A., Tatsumisago M. (2019). J. Mater. Chem. A.

[cit24] Yubuchi S., Nakamura W., Bibienne T., Rousselot S., Taylor L. W., Pasquali M., Dollé M., Sakuda A., Hayashi A., Tatsumisago M. (2019). J. Power Sources.

[cit25] Yubuchi S. , Uematsu M., Sakuda A., Hayashi A. and Tatsumisago M., 255^th^ ACS National Meeting & Exposition, 2018

[cit26] Chida S., Miura A., Rosero-Navarro N. C., Higuchi M., Phuc N. H. H., Muto H., Matsuda A., Tadanaga K. (2018). Ceram. Int..

[cit27] Uematsu M., Yubuchi S., Noi K., Sakuda A., Hayashi A., Tatsumisago M. (2018). Solid State Ionics.

[cit28] Uematsu M., Yubuchi A., Tsuji F., Sakuda A., Hayashi A., Tatsumisago M. (2019). J. Power Sources.

[cit29] Wan H., Mwizerwa J. P., Qi X., Xu X., Li H., Zhang Q., Cai L., Hu Y.-S., Yao X. (2018). ACS Appl. Mater. Interfaces.

[cit30] Yubuchi S., Ito A., Masuzawa N., Sakuda A., Hayashi A., Tatsumisago M. J. Mater. Chem. A.

[cit31] Banerjee A., Park K. H., Heo J. W., Nam Y. J., Moon C. K., Oh S. M., Hong S.-T., Jung Y. S. (2016). Angew. Chem., Int. Ed..

[cit32] Wan H., Mwizerwa J. P., Qi X., Liu X., Xu X., Li H., Hu Y.-S., Yao X. (2018). ACS Nano.

[cit33] Wang Y., Liu Z., Zhu X., Tang Y., Huang F. (2013). J. Power Sources.

[cit34] Park K. H., Oh D. Y., Choi Y. E., Nam Y. J., Han L., Kim J.-Y., Xin H., Lin F., Oh S. M., Jung Y. S. (2016). Adv. Mater..

[cit35] Choi Y. E., Park K. H., Kim D. H., Oh D. Y., Kwak H. R., Lee Y.-G., Jung Y. S. (2017). ChemSusChem.

[cit36] Mizuno F., Hayashi A., Tadanaga K., Tatsumisago M. (2005). Adv. Mater..

[cit37] Hayashi A., Ohtomo T., Mizuno F., Tadanaga K., Tatsumisago M. (2003). Electrochem. Commun..

[cit38] Izumi F., Momma K. (2007). Solid State Phenom..

[cit39] Rigaku J., 2012, 28, 29 30

[cit40] Weber T., Muijsers J. C., Niemantsverdriet J. W. (1995). J. Phys. Chem..

[cit41] Tsukasaki H., Mori S., Otoyama M., Yubuchi S., Asano T., Tanaka Y., Ohno T., Mori S., Hayashi A., Tatsumisago M. (2018). Sci. Rep..

[cit42] Lábár J. L. (2005). Ultramicroscopy.

[cit43] Lábár J. L. (2008). Microsc. Microanal..

[cit44] Lábár J. L. (2009). Microsc. Microanal..

[cit45] Lábár J. L., Adamik M., Barna B. P., Czigány Z., Fogarassy Z., Horváth Z. E., Geszti O., Misják F., Morgiel J., Radnóczi G., Sáfrán G., Székely L., Szüts T. (2012). Microsc. Microanal..

[cit46] Tsukasaki H., Mori S., Shiotani S., Yamamura H., Iba H. (2017). J. Power Sources.

[cit47] Tanibata N., Tsukasaki H., Deguchi M., Mori S., Hayashi A., Tatsumisago M. (2017). Solid State Ionics.

[cit48] Momma K., Izumi F. (2011). J. Appl. Crystallogr..

