# Supplementary material for: Quantitative analysis of crystallinity in an argyrodite sulfide-based solid electrolyte synthesized via solution processing
Source: RSC Adv. 2019 May 8;9(25):14465–71. doi: 10.1039/c9ra00949c (PMC9064144; doi:10.1039/c9ra00949c)
Supplement: RA-009-C9RA00949C-s001 [file RA-009-C9RA00949C-s001.pdf]

Electronic Supplementary Material (ESI) for RSC Advances.
This journal is © The Royal Society of Chemistry 2019

Quantitative Analysis of Crystallinity in an Argyrodite Sulfide-Based Solid Electrolyte Synthesized *via* a Solution

SUPPORTING INFORMATION

So Yubuchi [a], Hirofumi Tsukasaki [b], Atsushi Sakuda [a], Shigeo Mori [b], Akitoshi Hayashi [a], and Masahiro Tatsumisago [a]*

a. Department of Applied Chemistry, Graduate School of Engineering, Osaka Prefecture University, 1-1, Gakuen-cho, Naka-ku, Sakai, Osaka 599-8531, Japan

b. Department of Materials Science, Graduate School of Engineering, Osaka Prefecture University, 1-1, Gakuen-cho, Naka-ku, Sakai, Osaka 599-8531, Japan

***Corresponding author:**

Akitoshi Hayashi (Professor)

Tel.: +81-72-2549331; Fax.: +81-72-2549334

E-mail address: hayashi@chem.osakafu-u.ac.jp

Mutual confirmation of the accuracy in a WPPF technique and TEM observation

The accuracy of the WPPF technique, which can be used to calculate the amount ratios of the crystals, was investigated. Amorphous Li_3PS_4 and crystalline γ-Li_3PS_4 were mixed at arbitrary ratios, and a linear relationship between the amount ratios of the γ-Li_3PS_4 crystals calculated from the WPPF technique and the mixed ratios was confirmed. Amorphous Li_3PS_4 and γ-Li_3PS_4 crystals were prepared based on previous reports.[1,2] Figure S9 (a) shows XRD patterns of the amorphous Li_3PS_4, γ-Li_3PS_4 crystal, and A_2O_3 crystal. Figure S9 (b) shows XRD patterns of the mixtures of the amorphous Li_3PS_4, γ-Li_3PS_4 crystal, and A_2O_3 crystal. The mixed weight ratios of the amorphous Li_3PS_4 and γ-Li_3PS_4 crystal were x:100–x (x = 0, 50, 80, 100). Figure S9 (c) presents the relationship between the amount ratios of the γ-Li_3PS_4 crystals calculated from the WPPF technique and the mixed ratios. The accuracy of the WPPF technique was confirmed to be high because of their reasonable correlation. The amount ratio of the γ-Li_3PS_4 crystal was 80%. This result agrees with previous reports where the crystallinities were 75 vol.% or less when the sulfide electrolytes such as the $Li_{10}GeP_2S_{12}$ and Li_3PS_4 glass-ceramic were heated at high temperatures.[3,4]

TEM observation of the γ-Li_3PS_4 crystal was also conducted. Figure S10 (a–d) shows the BF image, ED pattern, DF images, and superposed DF image, respectively. The main crystalline phase was identified as the γ-Li_3PS_4 crystal. From the superimposed DF images, the volume ratio of the crystallized region was calculated to be 74%. The ratio calculated from the TEM observation is lower than that obtained from the WPPF technique because the volume ratios of the crystalline are provided from the two-dimensional images. However, the results are considered sufficient to ensure the validity of the WPPF technique.

References

1. F. Mizuno, A. Hayashi, K. Tadanaga and M. Tatsumisago, *Advanced Mater.,* 2005, 17(7), 918–921.
2. K. Homma, M. Yonemura, T. Kobayashi, M. Nagao, M. Hirayama and R. Kanno, *Solid State Ionics*, 2011, 182, 53–58.
3. H. Tsukasaki, H. Mori, M. Deguchi, S. Mori, A. Hayashi and M. Tatsumisago, *Solid State Ionic*s, 2017, 311, 6–13.
4. H. Tsukasaki, S. Mori, S. Shiotani, H. Yamamura and H. Iba, *J. Power Source*s, 2017, 369, 57–64.

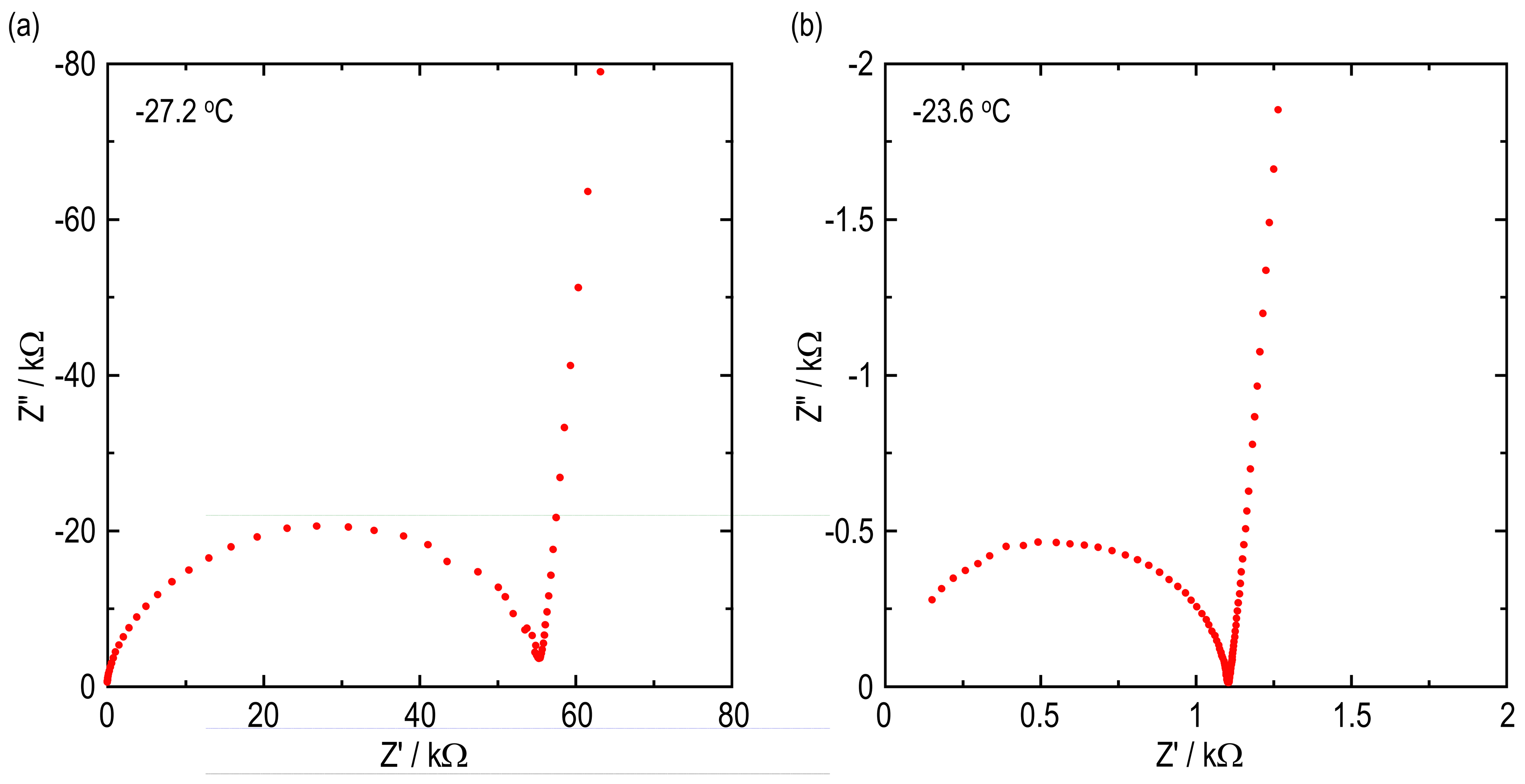

Figure S1 Nyquist plots of the argyrodite electrolytes prepared by heating at (a) 150 °C and (b) 400 °C. The impedance measurements were conducted at low temperatures below -20 °C.

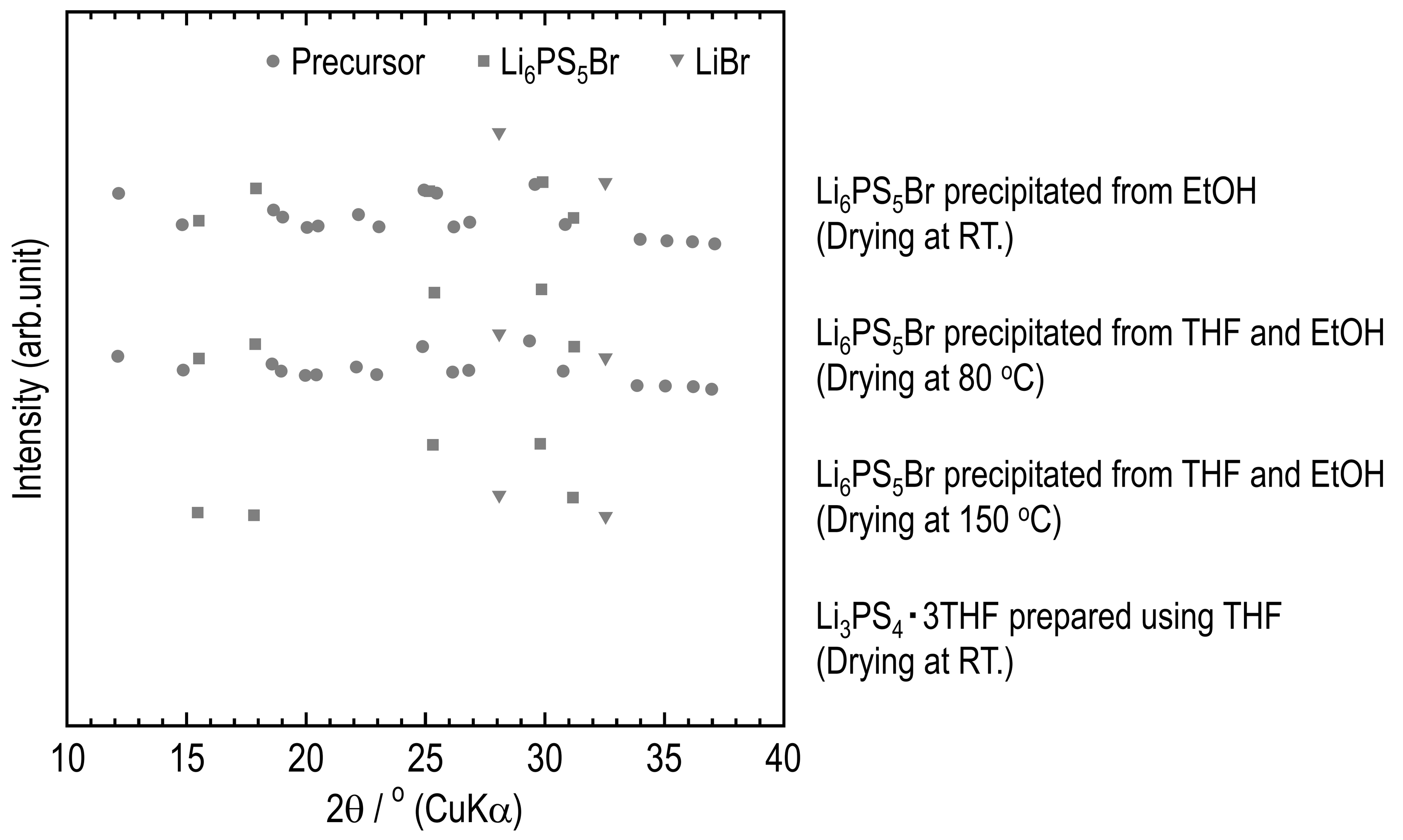

Figure S2 XRD patterns of Li_6PS_5Br precipitated from the EtOH precursor solution by drying at RT, Li_6PS_5Br precipitated from the THF and EtOH precursor solutions by drying at 80 °C and 150 °C, and Li_3PS_4·3THF prepared by drying at RT.

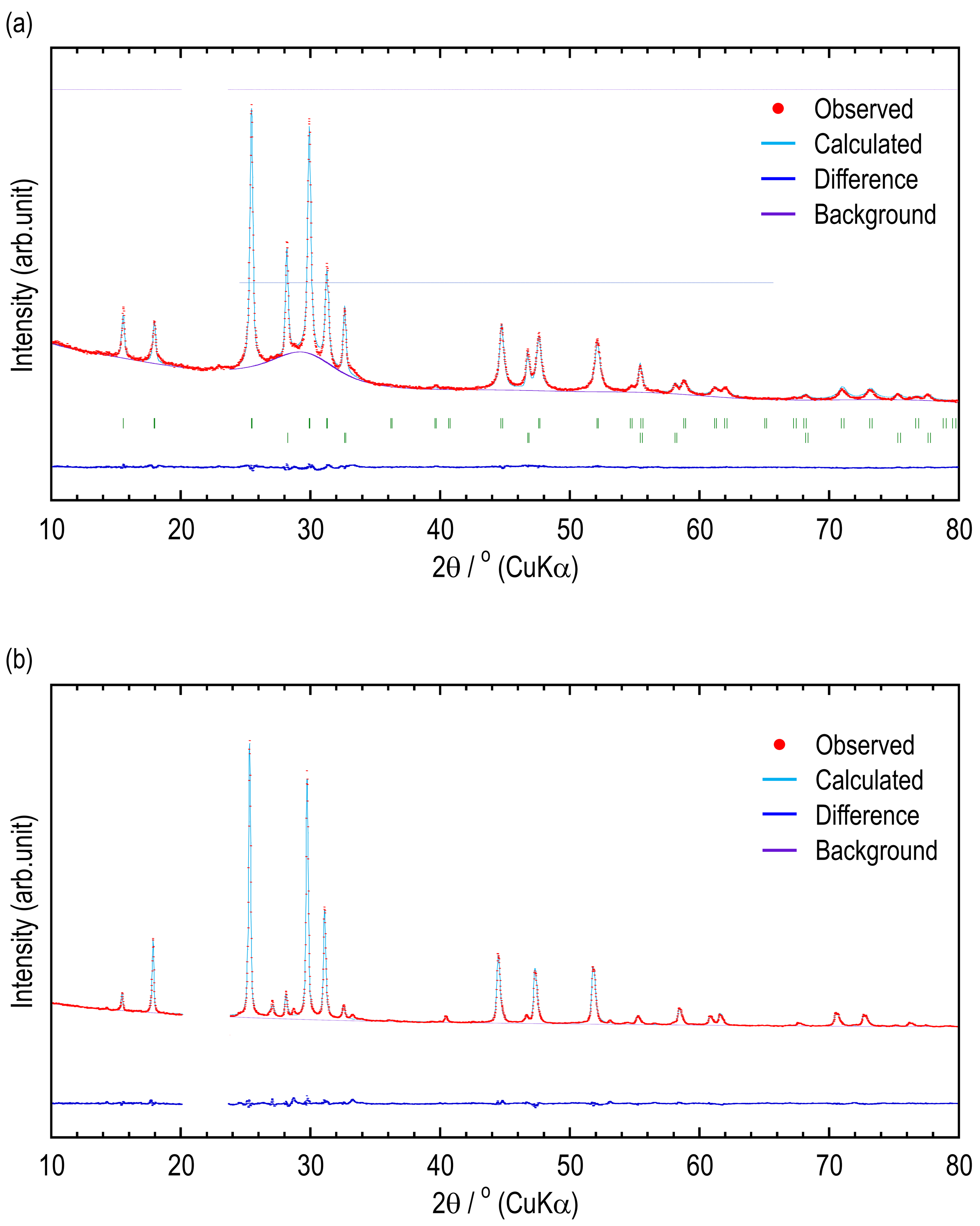

Figure S3 Rietveld refinement profiles of powder XRD data for the argyrodite electrolytes heated at (a) 150 °C and (b) 400 °C. The data were recorded at room temperature. Red dots and light blue lines denote the observed and calculated XRD patterns, respectively. The green sticks mark the position of the reflections for Li_6PS_5Br, LiBr, and Li_2S. The difference between the observed and calculated patterns is indicated by the blue lines. The background is represented by the purple lines.

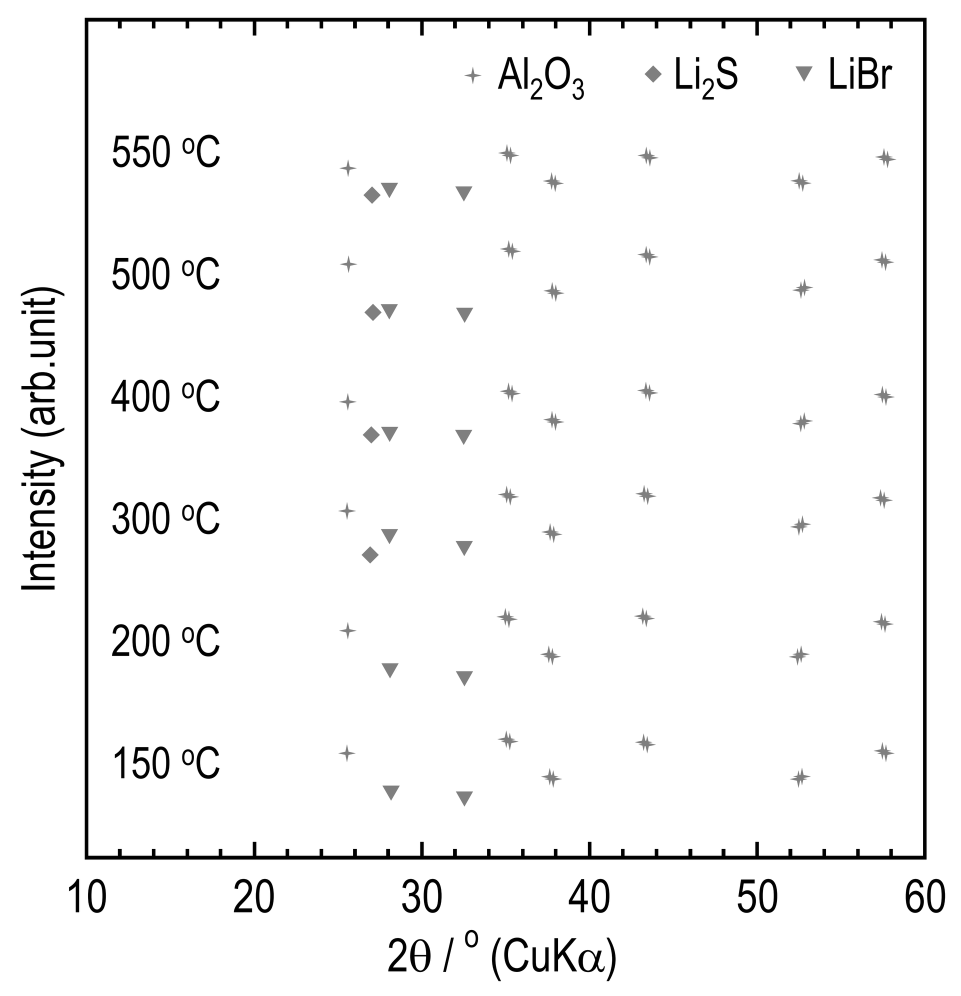

Figure S4 XRD patterns of the mixtures of Al_2O_3 crystals and the argyrodite electrolytes.

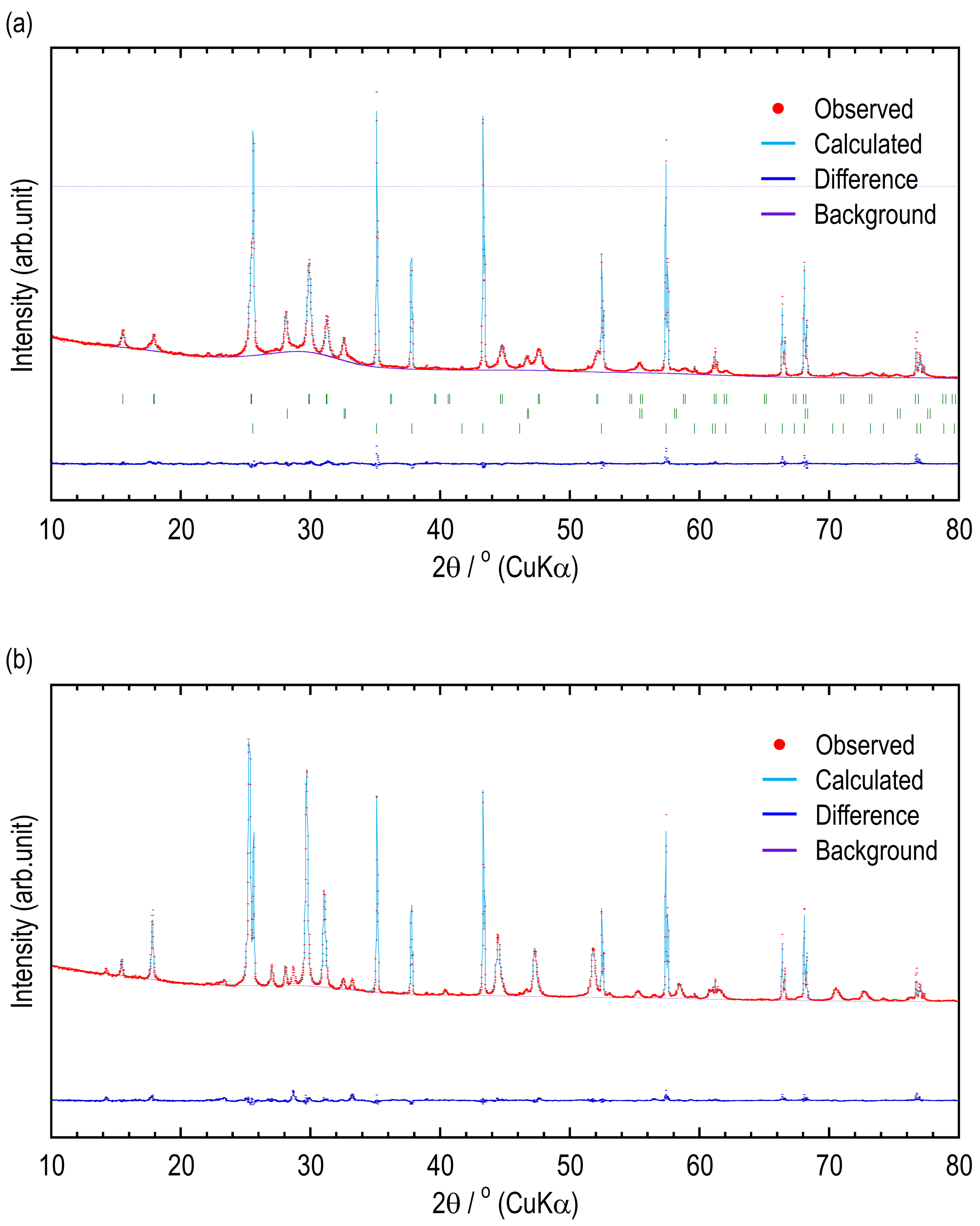

Figure S5 Rietveld refinement profiles of powder XRD data for the mixtures of Al_2O_3 crystals and the argyrodite electrolytes heated at (a) 150 °C and (b) 400 °C. The data were recorded at room temperature. Red dots and light blue lines denote the observed and calculated XRD patterns, respectively. The green sticks mark the position of the reflections for Li_6PS_5Br, LiBr, Li_2S, and Al_2O_3. The difference between the observed and calculated patterns is indicated by the blue lines. The background is represented by the purple lines.

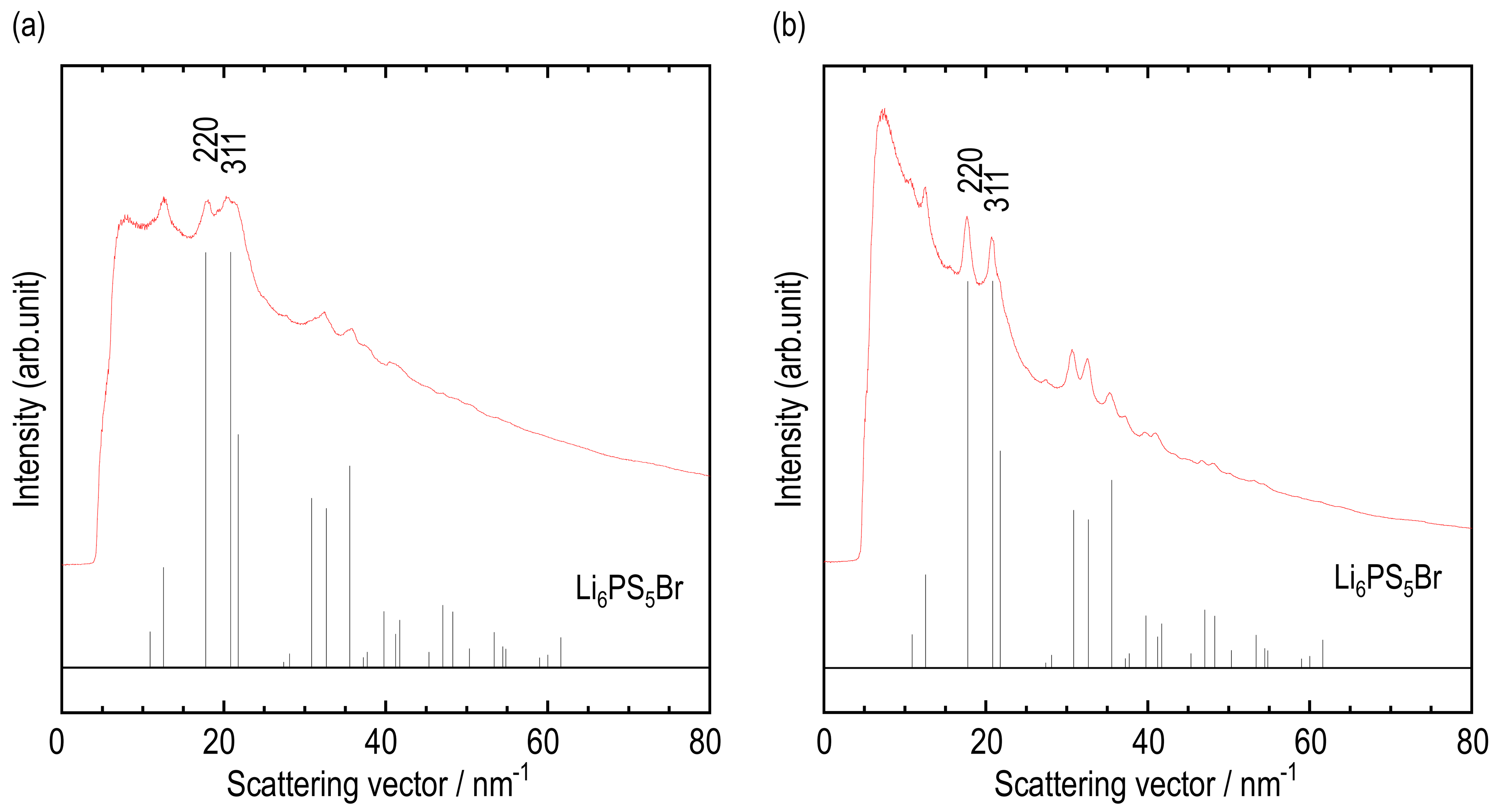

Figure S6 Intensity profiles of the ED patterns of the argyrodite electrolytes heated at (a) 150 °C and (b) 400 °C with XRD card data of Li_6PS_5Br.

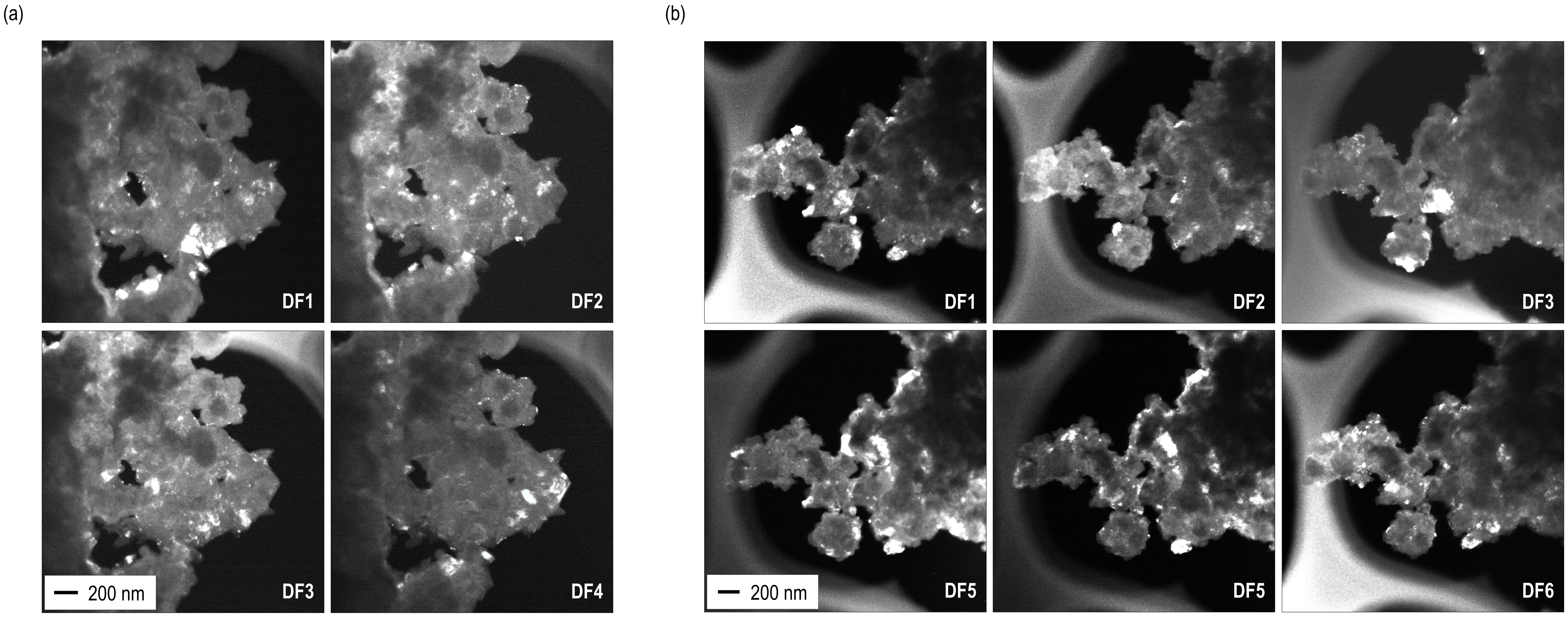

Figure S7 DF images taken of all the diffraction spots in the ED patterns of the argyrodite electrolytes heated at (a) 150 ^{o}C and (b) 400 ^{o}C.

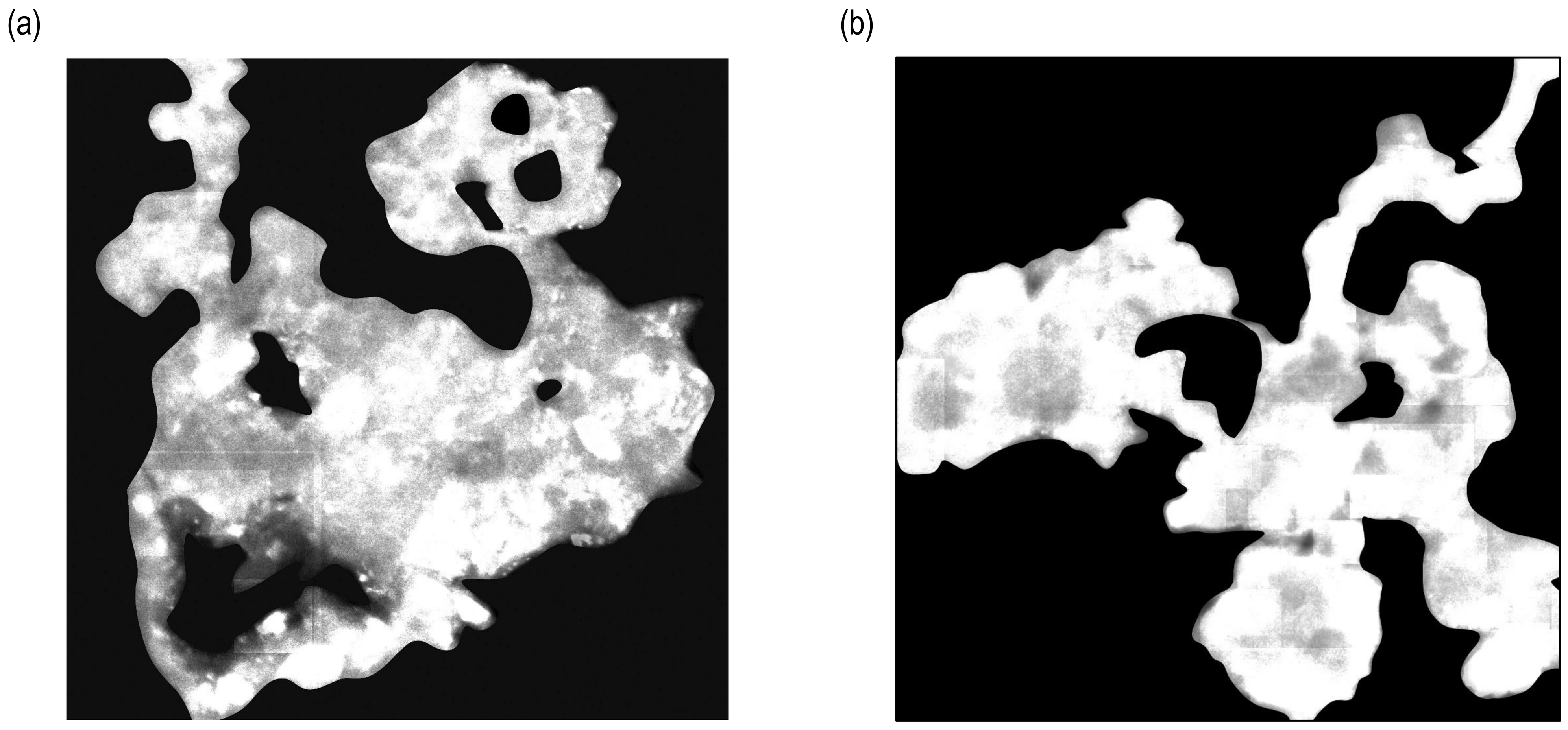

Figure S8 DF images of the region used to determine the amount ratios of the crystals in the argyrodite electrolytes heated at (a) 150 ^{o}C and (b) 400 ^{o}C. Bright-contrast regions are the areas including the crystallites. The black-color regions correspond to the vacuum region and the thick electrolyte regions are excluded from the calculations.

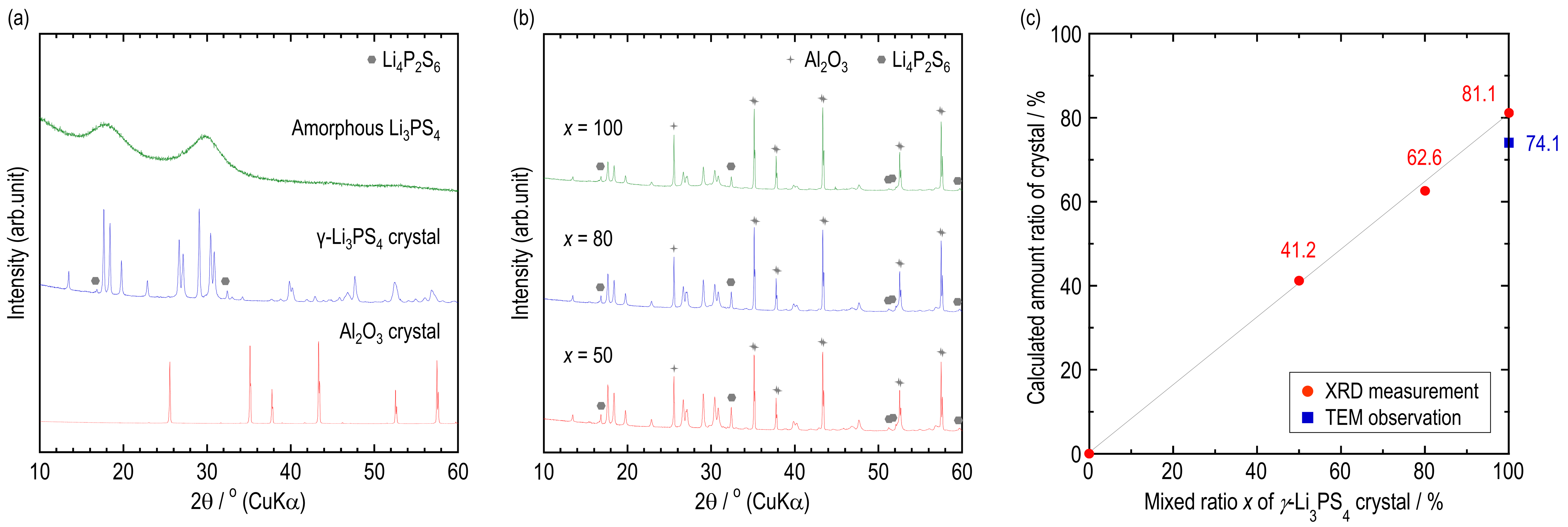

Figure S9 (a) XRD patterns of the amorphous Li_3PS_4, the γ-Li_3PS_4 crystal, and A_2O_3 crystal. (b) XRD patterns of the mixtures of the amorphous Li_3PS_4, the γ-Li_3PS_4 crystals, and A_2O_3 crystals. The mixed weight ratios of the amorphous Li_3PS_4 and the γ-Li_3PS_4 crystals were x:100–x (x = 0, 50, 80, 100). (c) Relationship between the amount ratios of the γ-Li_3PS_4 crystals calculated from the WPPF technique and the mixed ratios of the γ-Li_3PS_4 crystals. The amount ratio of the γ-Li_3PS_4 crystal observed from a TEM observation is also shown. The numbers in the figure represent the amount ratios of the crystal obtained from the XRD and TEM measurements.

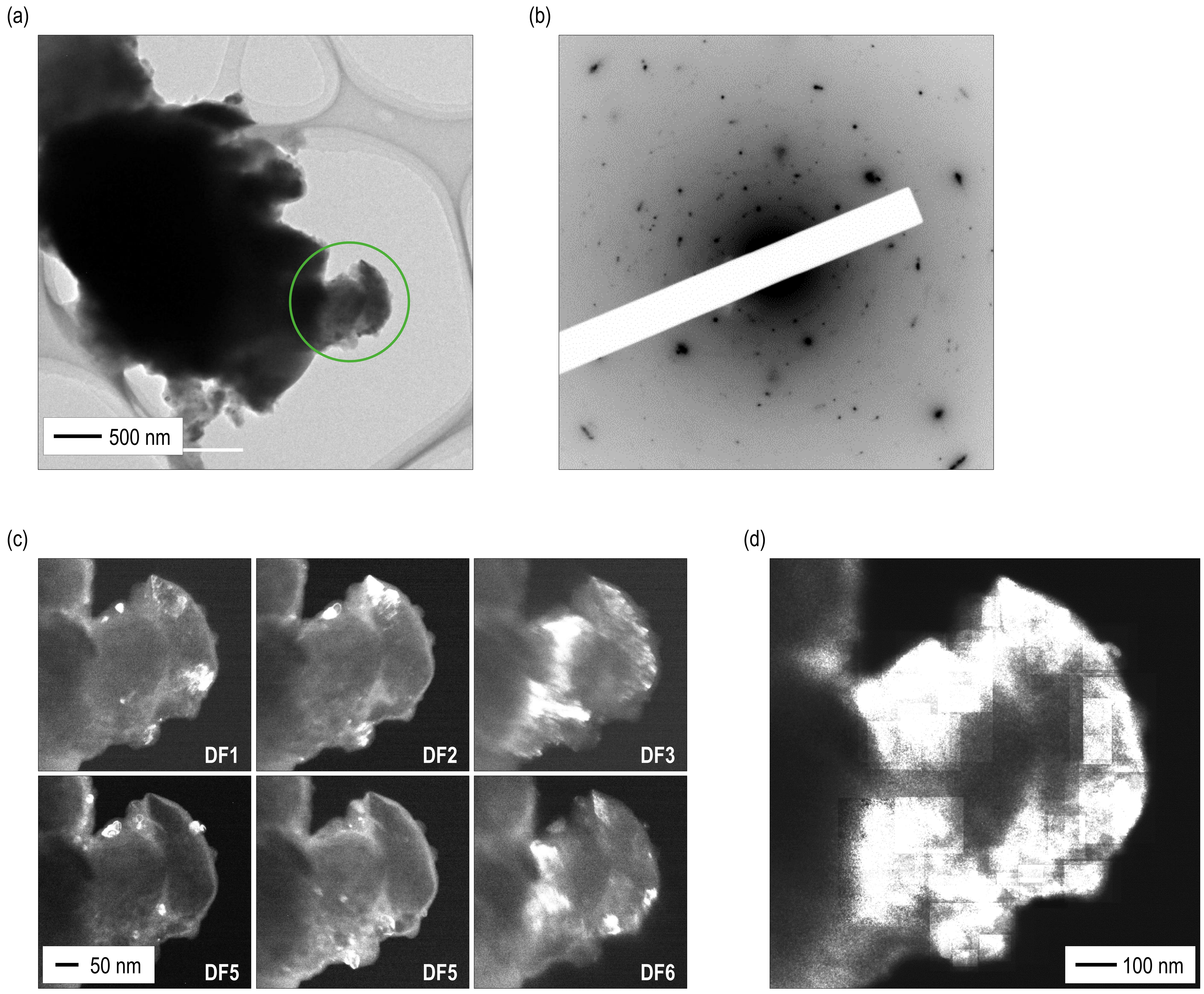

Figure S10 (a) BF image, (b) ED pattern, (c) DF images, and (d) superposed DF images of the γ-Li_3PS_4 crystal taken from a TEM observation.

Table S1 Crystallographic data for Li_6PS_5Br prepared with heat treatment at 150 ^{o}C.

Crystal system	Cubic	Lattice parameter	a = 9.9220(3) Å
Space group	$F\bar{4}3m$ (no. 216)	Volume	V = 976.7(1) Å^3

Atom	Wyckoff site	g	x	y	z	U / Å^2
Li	48*h*	1/2	0.314(2)	0.019(2)	= 1 - *x*(Li)	0.0633
P	4*b*	1.0	0.0	0.0	1/2	0.0253
S1	4*a*	= 1 - *g*(Br1)	0.0	0.0	1.0	0.0253
S2	4*d*	= *g*(Br1)	1/4	1/4	3/4	0.0253
S3	16*e*	1.0	0.1151(2)	= -*x*(S3)	= 0.5 + *x*(S3)	0.0253
Br1	4*a*	0.523(6)	0.0	0.0	1.0	0.0253
Br2	4*d*	= 1 - *g*(Br1)	1/4	1/4	3/4	0.0253

*R_{wp} = 3.772, R_F = 5.724, R_B = 6.127, $S = R_{wp}/R_e$ = 2.3242

Table S2 Crystallographic data for Li_6PS_5Br prepared with heat treatment at 200 ^{o}C.

Crystal system	Cubic	Lattice parameter	a = 9.9350(9) Å
Space group	$F\bar{4}3m$ (no. 216)	Volume	V = 980.6(1) Å^3

Atom	Wyckoff site	g	x	y	z	U / Å^2
Li	48*h*	1/2	0.294(1)	0.010(4)	= 1 - *x*(Li)	0.0633
P	4*b*	1.0	0.0	0.0	1/2	0.0253
S1	4*a*	= 1 - *g*(Br1)	0.0	0.0	1.0	0.0253
S2	4*d*	= *g*(Br1)	1/4	1/4	3/4	0.0253
S3	16*e*	1.0	0.1157(2)	= -*x*(S3)	= 0.5 + *x*(S3)	0.0253
Br1	4*a*	0.667(6)	0.0	0.0	1.0	0.0253
Br2	4*d*	= 1 - *g*(Br1)	1/4	1/4	3/4	0.0253

*R_{wp} = 3.688, R_F = 3.900, R_B = 6.9897, $S = R_{wp}/R_e$ = 2.2444

Table S3 Crystallographic data for Li_6PS_5Br prepared with heat treatment at 300 °C.

Crystal system	Cubic	Lattice parameter	a = 9.9590(6) Å
Space group	$F\bar{4}3m$ (no. 216)	Volume	V = 987.7(1) Å^3

Atom	Wyckoff site	g	x	y	z	U / Å^2
Li	48*h*	1/2	0.292(1)	0.020(2)	= 1 - *x*(Li)	0.0633
P	4*b*	1.0	0.0	0.0	1/2	0.0253
S1	4*a*	= 1 - *g*(Br1)	0.0	0.0	1.0	0.0253
S2	4*d*	= *g*(Br1)	1/4	1/4	3/4	0.0253
S3	16*e*	1.0	0.1178(1)	= -*x*(S3)	= 0.5 + *x*(S3)	0.0253
Br1	4*a*	0.726(3)	0.0	0.0	1.0	0.0253
Br2	4*d*	= 1 - *g*(Br1)	1/4	1/4	3/4	0.0253

*R_{wp} = 3.373, R_F = 0.884, R_B = 2.262, $S = R_{wp}/R_e$ = 2.0523

Table S4 Crystallographic data for Li_6PS_5Br prepared with heat treatment at 400 °C.

Crystal system	Cubic	Lattice parameter	a = 9.9762(2) Å
Space group	$F\bar{4}3m$ (no. 216)	Volume	V = 994.89(4) Å^3

Atom	Wyckoff site	g	x	y	z	U / Å^2
Li	48*h*	1/2	0.3062(9)	0.013(1)	= 1 - *x*(Li)	0.0633
P	4*b*	1.0	0.0	0.0	1/2	0.0253
S1	4*a*	= 1 - *g*(Br1)	0.0	0.0	1.0	0.0253
S2	4*d*	= *g*(Br1)	1/4	1/4	3/4	0.0253
S3	16*e*	1.0	0.1184(1)	= -*x*(S3)	= 0.5 + *x*(S3)	0.0253
Br1	4*a*	0.712(2)	0.0	0.0	1.0	0.0253
Br2	4*d*	= 1 - *g*(Br1)	1/4	1/4	3/4	0.0253

*R_{wp} = 3.657, R_F = 1.026, R_B = 1.751, $S = R_{wp}/R_e$ = 2.2491

Table S5 Crystallographic data for Li_6PS_5Br prepared with heat treatment at 500 °C.

Crystal system	Cubic	Lattice parameter	a = 9.9777(1) Å
Space group	$F\bar{4}3m$ (no. 216)	Volume	V = 993.33(3) Å^3

Atom	Wyckoff site	*g*	*x*	*y*	*z*	*U* / Å^2
Li	48*h*	1/2	0.3001(9)	0.000(2)	= 1 - *x*(Li)	0.0633
P	4*b*	1.0	0.0	0.0	1/2	0.0253
S1	4*a*	= 1 - *g*(Br1)	0.0	0.0	1.0	0.0253
S2	4*d*	= *g*(Br1)	1/4	1/4	3/4	0.0253
S3	16*e*	1.0	0.1175(1)	= -*x*(S3)	= 0.5 + *x*(S3)	0.0253
Br1	4*a*	0.787(3)	0.0	0.0	1.0	0.0253
Br2	4*d*	= 1 - *g*(Br1)	1/4	1/4	3/4	0.0253

*R_{wp} = 4.100, R_F = 2.718, R_B = 4.589, $S = R_{wp}/R_e$ = 2.4451

Table S6 Crystallographic data for Li_6PS_5Br prepared with heat treatment at 550 °C.

Crystal system	Cubic	Lattice parameter	a = 9.9674(1) Å
Space group	$F\bar{4}3m$ (no. 216)	Volume	V = 990.26(2) Å^3

Atom	Wyckoff site	*g*	*x*	*y*	*z*	*U* / Å^2
Li	48*h*	1/2	0.3012(9)	0.008(1)	= 1 - *x*(Li)	0.0633
P	4*b*	1.0	0.0	0.0	1/2	0.0253
S1	4*a*	= 1 - *g*(Br1)	0.0	0.0	1.0	0.0253
S2	4*d*	= *g*(Br1)	1/4	1/4	3/4	0.0253
S3	16*e*	1.0	0.1174(1)	= -*x*(S3)	= 0.5 + *x*(S3)	0.0253
Br1	4*a*	0.698(2)	0.0	0.0	1.0	0.0253
Br2	4*d*	= 1 - *g*(Br1)	1/4	1/4	3/4	0.0253

*R_{wp} = 3.979, R_F = 3.255, R_B = 4.946, $S = R_{wp}/R_e$ = 2.4549
